# Stability of Boron Nitride Nanosphere Dispersions in the Presence of Polyelectrolytes

**DOI:** 10.1021/acs.langmuir.1c00656

**Published:** 2021-04-20

**Authors:** Lívia Vásárhelyi, Tímea Hegedűs, Szilárd Sáringer, Gergő Ballai, István Szilágyi, Zoltán Kónya

**Affiliations:** † Interdisciplinary Excellence Center, Department of Applied and Environmental Chemistry, University of Szeged, Szeged H-6720, Hungary; ‡ MTA-SZTE Lendület Biocolloids Research Group, Interdisciplinary Excellence Center, Department of Physical Chemistry and Materials Science, University of Szeged, Szeged H-6720, Hungary; § MTA-SZTE Reaction Kinetics and Surface Chemistry Research Group, Szeged H-6720, Hungary

## Abstract

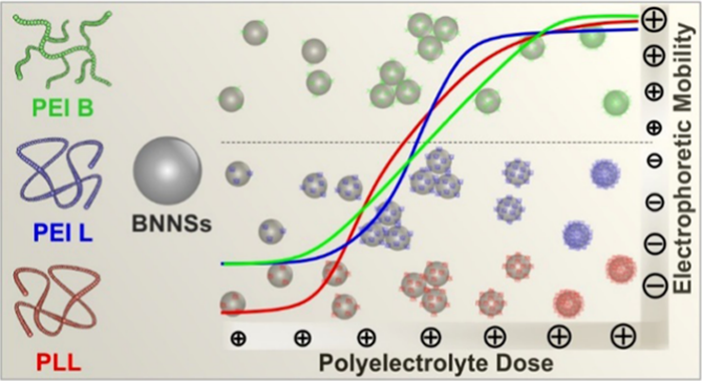

Boron nitride nanospheres (BNNSs) were functionalized with polyelectrolytes. The effect of the polyelectrolyte dose and ionic strength on the charging and aggregation properties was investigated. At appropriate polyelectrolyte doses, charge neutralization occurred, whereas by increasing the dose, charge reversal was observed. The complete coating of the particles was indicated by a plateau in the ζ-potential values, which do not change significantly beyond the dose corresponding to the onset of such a plateau. The dispersions were highly aggregated around the charge neutralization point, while at lower or higher doses, the particles were stable. The salt-induced aggregation experiments revealed that the polyelectrolyte coatings contribute to the colloidal stability of the particles, namely, the critical coagulation concentrations deviated from the one determined for bare BNNSs. The presence of electrostatic and steric interparticle forces induced by the adsorbed polyelectrolyte chains was assumed. The obtained results confirm that the comprehensive investigation of the colloidal stability of BNNS particles is crucial to design stable or unstable dispersions and that polyelectrolytes are suitable agents for both stabilization and destabilization of BNNS dispersions, depending on the purpose of their application.

## Introduction

Various engineered inorganic nanomaterials have been utilized for medical applications, including cancer treatment^1^ and the combination of cancer therapy and diagnosis, referred to as theranostics.^2^ Among them, boron nitride (BN) has recently gained considerable interest since its different allotropes are structural analogues to carbon species.^3^ They cannot be found in nature; therefore, it is necessary to synthesize them in different crystalline forms^4^ with stacked layers of hexagonal (h-BN) or rhombohedral geometries; cubic and wurtzite types are also common.^5^ They can form different nanostructures such as nanotubes or nanosheets.^6^ Besides, nanospheres are also of great interest.^7^

The structure of h-BN is composed of an equal number of boron (B) and nitrogen (N) atoms forming a honeycomb-like structure, where the atoms are connected with strong covalent bonds.^8^ However, the electron distribution between the atoms is different, as N possesses a higher electronegativity, therefore attracting the electrons more strongly.^9^ This results in distinct electronic, thermal, and optical characteristics compared to carbon materials^10−12^ and improved chemical stability.^13^ BN nanostructures have been proposed to apply in catalysis^14−16^ or to improve the properties of composite materials.^17,18^ A very important feature of theirs is biocompatibility;^19^ BN nanoparticles are not cytotoxic;^20^ therefore, they can be used in various biomedical applications. BN nanospheres (BNNSs) strongly interact with cells,^21^ which is important in terms of biorelated processes. These include effective drug delivery systems^20−23^ and combined chemotherapy and radiation therapy.^24,25^ Besides, they were suggested as a boron reservoir in boron neutron capture therapy (BNCT)^26,27^ because numerous boron atoms are accumulated in a small volume. With surface functionalization of the BNNS, it is possible to improve the biocompatibility, while the colloidal properties become tunable.

Polyelectrolytes (PE) have demonstrated great efficiency toward stabilization or destabilization of particle dispersions in aqueous media^28^ and have been proposed to tune charging and aggregation features of various colloidal and nanoparticles.^29−32^ The colloidal stability is particularly important in the case of BN nanomaterials, as they are often expected to be used in heterogeneous systems and physiological environments like bloodstream. As dispersions of BNNSs tend to aggregate, suitable methods must be employed to enhance the colloidal stability^20^ since poorly dispersed nanomaterials do not exhibit the expected features.^33^ The adsorption of PEs on oppositely charged particle surfaces is strong and thus significantly changes the charging behavior. Once particles are dispersed in PE solutions, this issue must be carefully considered to understand and control the properties of particle suspensions.^28^ The noncovalent functionalization of boron nitride nanoparticles with PEs turned out to be more effective compared to chemical functionalization, due to the high chemical stability of this material.^34^ Weak PEs, in which charge densities vary with respect to the pH and ionic strength of the solutions,^35^ have been reported for the modification of nanostructures to improve their dispersity and stability in aqueous samples.^36^

There are examples in the literature dealing with the functionalization of the BNNS with PEs. Nanoparticles modified with poly(allylamine)-citraconic anhydride can undergo charge reversal by changing the environmental pH; therefore, long-term stability in the blood is ensured.^24^ Polyethyleneimine (PEI) is a cationic PE, rich in amine groups, thus Lewis acid–base interactions with the particles can be observed.^18^ At low and intermediate pH values, it possesses positive charge density in aqueous solution due to the presence of protonated primary and secondary amino groups.^35^ Therefore, PEI is able to absorb on the negatively charged BN surface through relatively strong electrostatic interactions, resulting in complexes with a strong positive charge.^17,23^ PEI has been used as a coating material for various inorganic nanoparticles.^36^ It can also be used for the simultaneous exfoliation and functionalization of BN, significantly improving their dispersibility and colloidal stability in water.^18,37^ BNNTs have been wrapped with PEI^38,39^ and have shown acceptable cytocompatibility when applied at low concentrations. Branched PEI, besides the already mentioned electrostatic interactions, can contribute to the stabilization of the dispersions by steric repulsion between like-charged particles.^23^ Polydopamine and PEI have been used to improve the surface properties of composite materials.^17,40^ Poly-l-lysine (PLL) is another type of cationic PE of natural origin; it was also considered as a dispersing agent for BNNTs.^41,42^

As mentioned earlier, PEs have been applied as stabilizing agents for BNNSs in certain applications.^23,24,40^ Among them, PLL was also considered for surface functionalization of BN nanotubes.^41,42^ However, there is a lack of systematic studies on the charging properties and the colloidal stability of BNNS coated with PEs in the literature. Although the colloidal behavior of the BNNSs has been recently studied in our lab to give insights into the relationship between the surface charge and tendency for aggregation in the presence of salts,^43^ no similar studies have been published with BNNS–PE systems yet. Therefore, in the present study, electrophoretic mobility and dynamic light scattering (DLS) measurements were conducted to address this issue for aqueous dispersions of BNNS in the presence of linear and branched polyethyleneimine (PEI L and PEI B) as well as PLL. The PE concentration and the ionic strength were systematically changed, and surface charges and aggregation rates were determined. The results shed light on the possible stabilization or destabilization of the BNNSs, opening the road for different applications, where stable or unstable dispersions are desired.

## Experimental Methods

### Materials

BNNSs were synthesized by chemical vapor deposition using B(OMe)_3_ as the B source and NH_3_ gas as the N source, as described elsewhere.^43^ Ionic strength was adjusted by analytical grade NaCl purchased from VWR. PEI B was bought from Alfa Aesar (*M*_w_ = 10 000 g/mol) and PEI L (*M*_w_ = 5000 g/mol) and PLL (*M*_w_ = 15 000 g/mol) of analytical grade from Sigma-Aldrich and were used as received. All of the measurements were carried out at 25 °C and pH 7. Ultrapure water obtained from a VWR Purity TU+ machine was used for sample preparations. Water and NaCl solutions were filtered with a 0.1 μm syringe filter (Millex) to avoid dust contamination.

### Electrophoretic Mobility

Both electrophoretic and DLS measurements were carried out with a Zetasizer Nano Instrument (Malvern) equipped with a 4 mW He–Ne laser (633 nm wavelength). The electrophoretic mobilities (*u*) were converted to ζ-potentials (ζ) by the Smoluchowski equation as^44^
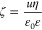
1where ε is the dielectric constant of the medium (78.5 at the respective temperature), η is the dynamic viscosity of water (8.9 × 10^–4^ Pa s), and ε_0_ is the dielectric permittivity of vacuum (8.9 × 10^–12^ F/m). For the determination of the ζ-potentials, 2 mL of dispersions were prepared by diluting the appropriate volume of particle dispersions or dispersions of PE-coated BNNS particles with an appropriate amount of water and salt solutions to obtain a final particle concentration of 5 mg/L. The samples were allowed to rest for 2 h at room temperature before each measurement, and the equilibration time in the device was 1 min. The reported ζ-potential values were the average of three individual measurements, and the average error was about 5%. The experiments were performed in disposable Zeta cells (Malvern).

### Dynamic Light Scattering

Time-resolved DLS measurements were performed to determine the apparent aggregation rate coefficients (*k*_app_) as^45,46^
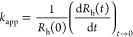
2where *R*_h_(0) is the hydrodynamic radius of the individual particles determined as 170 nm by DLS in stable suspensions and *t* is the time of the experiment. To measure the hydrodynamic radii, the correlation function was recorded for 20 s and time-resolved experiments lasted for 30 min. Greater apparent aggregation rate coefficients indicate unstable dispersions, in which rapid aggregation occurs. For each measurement, 2 mL of dispersions were prepared in the same way as described above for the electrophoretic measurements, but the DLS experiments were initiated by adding the appropriate volume of the particle stock dispersions leading to a final concentration of 5 mg/L. The samples were equilibrated for 60 s in the instrument. The colloidal stability of the suspensions was expressed in terms of the stability ratio (*W*) as^47,48^
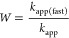
3where the fast condition indicates the diffusion-controlled aggregation of the particles in a 1 M NaCl solution. By considering eq 3, one can realize that a stability ratio of 1 is determined for fast particle aggregations occurred in unstable dispersions, where all particle collisions result in dimer formation. Consequently, higher values correspond to slower aggregation. This protocol led to a mean error of 5%.

### Fourier Transform Infrared (FTIR) Spectroscopy

The spectra of the bare and PE-coated BNNSs were recorded using a Bruker Vertex 70 FTIR device operating in a wavenumber range from 400 to 4000 cm^–1^, with a resolution of 4 cm^–1^ in transmission mode.

## Results and Discussion

The charging and aggregation properties of the BNNSs were investigated in the presence of different PEs and varying ionic strengths. Note that the experimental conditions (e.g., pH, PE dose range, ionic strength, and BNNS concentration) were the same in both electrophoretic and DLS measurements. Later, the effect of the PEs on the interparticle forces was interpreted.

### Characterization of the BNNSs

**Figure 1:**
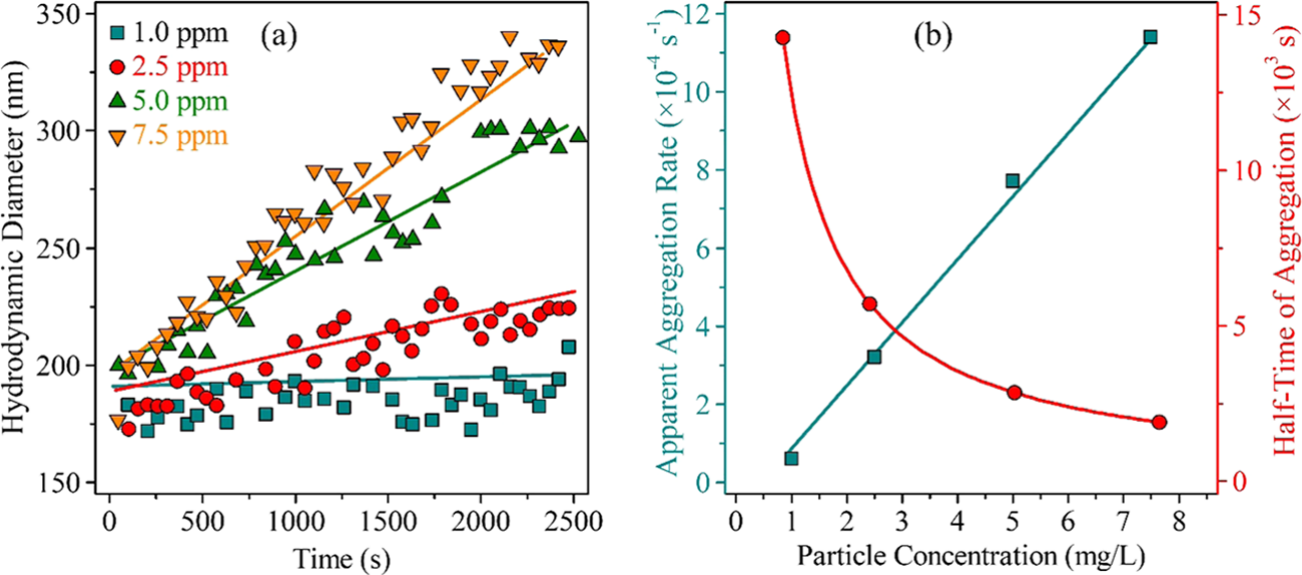
(a) Time-resolved DLS measurements at different particle concentrations at 1 M ionic strength. The solid lines are linear fits used to calculate the apparent aggregation rates in eq 2. (b) The calculated apparent aggregation rates and half-time of aggregation by eq 5 at different particle concentrations.

The detailed structural characterization of the BNNS particles can be found elsewhere.^43^ The particles show good crystallinity and high purity. Prior to the time-resolved DLS measurements, the experimental conditions such as the particle concentration was optimized. One must make a compromise that aggregation remains in the early stages, i.e., the increase in the hydrodynamic radius is linear with time and that the scattered intensity is high enough to perform reliable DLS measurements.^45,46^ Accordingly, time-resolved DLS experiments were performed in 1 M NaCl solutions at different particle concentrations (Figure 1a). At such a high salt concentration, the aggregation is merely driven by the diffusion of the particles. The calculated *k*_app_ data are given in Figure 1b.

When fast aggregation occurs, it is assumed that the diffusing particles do not interact during encounter but stick to each other. This model is equivalent to the diffusion-controlled fast aggregation process. It was also proved that the corresponding aggregation rate coefficient (*k*_s_) can be given as^47^
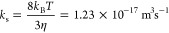
4where *T* is the absolute temperature and *k*_B_ is the Boltzmann constant. The calculated value was used to determine the half-time of aggregation (*T*_1/2_) (Figure 1b). This is the time interval, under which the initial concentration of the primary particles (*N*_0_) decreased by 50%. The value for *N*_0_ that is the initial particle concentration was calculated using the diameter of the particles measured by TEM (110 nm) and the density of h-BN (2.1 g/cm^3^).^33^ In the case of diffusion-controlled aggregation, the half-time of aggregation is given by the following equation^47^
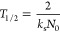
5

Based on these findings, a particle concentration of 5 mg/L was chosen to conduct further investigations due to the best compromise between the optimal half-time of aggregation and the intensity of the scattered light. The concentration-normalized fast apparent aggregation rate coefficient of dimer formation refers to the aggregation rate in unstable dispersions and was determined as 7.73 × 10^–18^ m^3^/s at a high salt concentration, where the electrostatic repulsion between the particles was screened by the salts and each collision of the particles resulted in the formation of aggregates.

**Figure 2:**
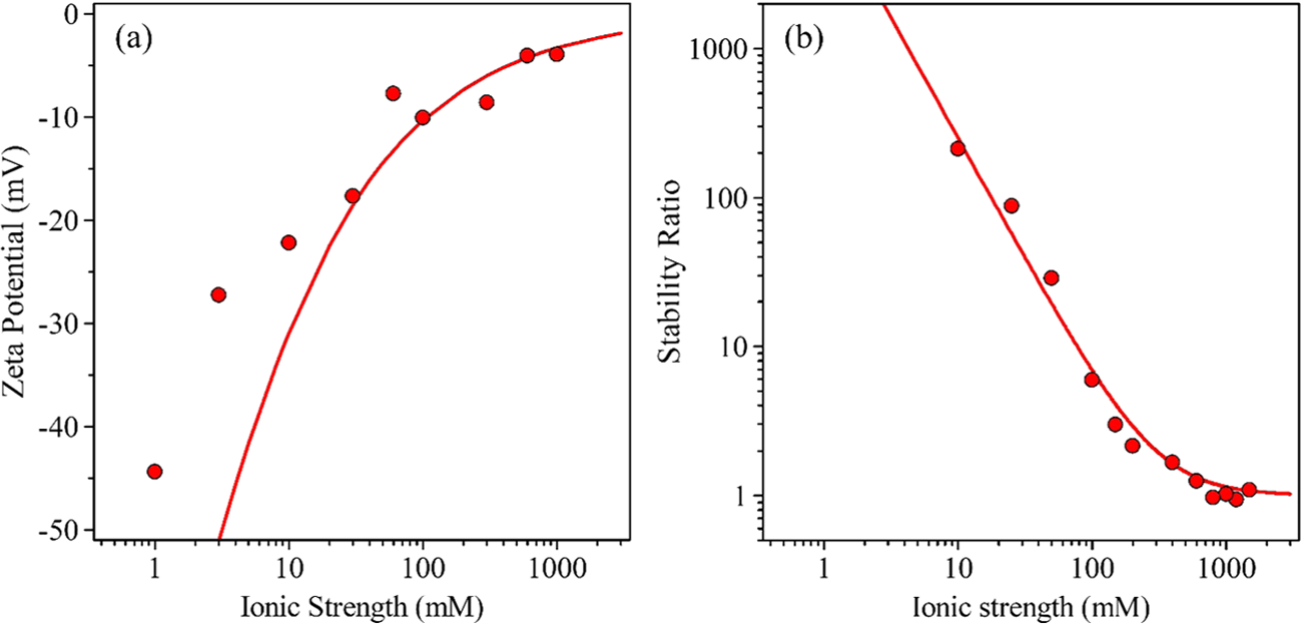
(a) ζ-potentials and (b) stability ratios of BNNS particles with varying ionic strength. The solid line in (a) was calculated using eq 6, while eq 8 was used in (b).

ζ-potential measurements at different ionic strengths (Figure 2a) were also carried out to reveal the charging mechanism of the bare nanoparticles. The ζ-potentials shift toward more positive values with increasing the salt concentration; however, overcharging of the particles cannot be observed even at high salt levels. Hence, at high ionic strengths, the dispersions are destabilized. The surface charge density at the slip plane (σ) was calculated according to the Grahame equation^49^ as
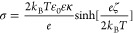
6where *e* is the elementary charge and κ is the inverse Debye length calculated using the ionic strength (*I*) as^44^
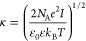
7where *N*_A_ is the Avogadro number. The inverse Debye length represents the contribution of all ionic species present in the dispersion for the quantitative description of the double layer.^44^ These equations served to fit the measured ζ-potentials at different ionic strengths to obtain the charge density at the slip plane according to eq 6. Note, that the Graham equation is valid only for higher ionic strengths over around 0.01 M. The value of σ in the case of the bare, uncoated BNNSs was determined as −7.5 mC/m^2^.

The stability of the bare particles in the presence of the NaCl electrolyte was determined by varying the ionic strength. From the stability ratio versus ionic strength plot (Figure 2b), it can be concluded that the calculated stability ratio decreased by increasing the ionic strength, and at a point, it reaches unity, meaning that the suspension is destabilized at a certain NaCl concentration. The transition between these sections is the so-called critical coagulation concentration (CCC) calculated as^48^
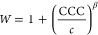
8where *c* is the molar concentration of the electrolyte and β is obtained from the change in the stability ratios with the salt concentration in the slow aggregation regime before the CCC as
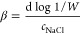
9

Equations 8 and 9 represent empirical formulas, and they serve to fit the stability ratio data at different salt concentrations to obtain the CCC values. The CCC value was determined for bare BNNS particles as 0.30 M. The above behavior is in line with the prediction of the theory by Derjaguin, Landau, Verwey, and Overbeek (DLVO) developed for the stability of charged colloidal particle dispersions in the presence of electrolytes.^50^

### Charging and Aggregation in the Presence of Polyelectrolytes

To assess the stability of BNNSs at different PE doses, aggregation rates and ζ-potentials were determined. In addition, these measurements also serve to explore the mechanism of particle functionalization with the PEs, as they can be effectively used to determine the PE dose needed to completely coat the surface of BNNSs. NaCl was added to adjust the background electrolyte at 0.001 M. Note that under these conditions, the nanoparticles were negatively charged (see ζ-potentials in Figure 2a), owing to the deprotonation of the surface N–O–H and B–O–H functional groups in the aqueous solutions.^17^ Therefore, the sign of the PE charge is opposite to the surface charge; thus, the adsorption is driven mainly by electrostatic forces.

**Figure 3:**
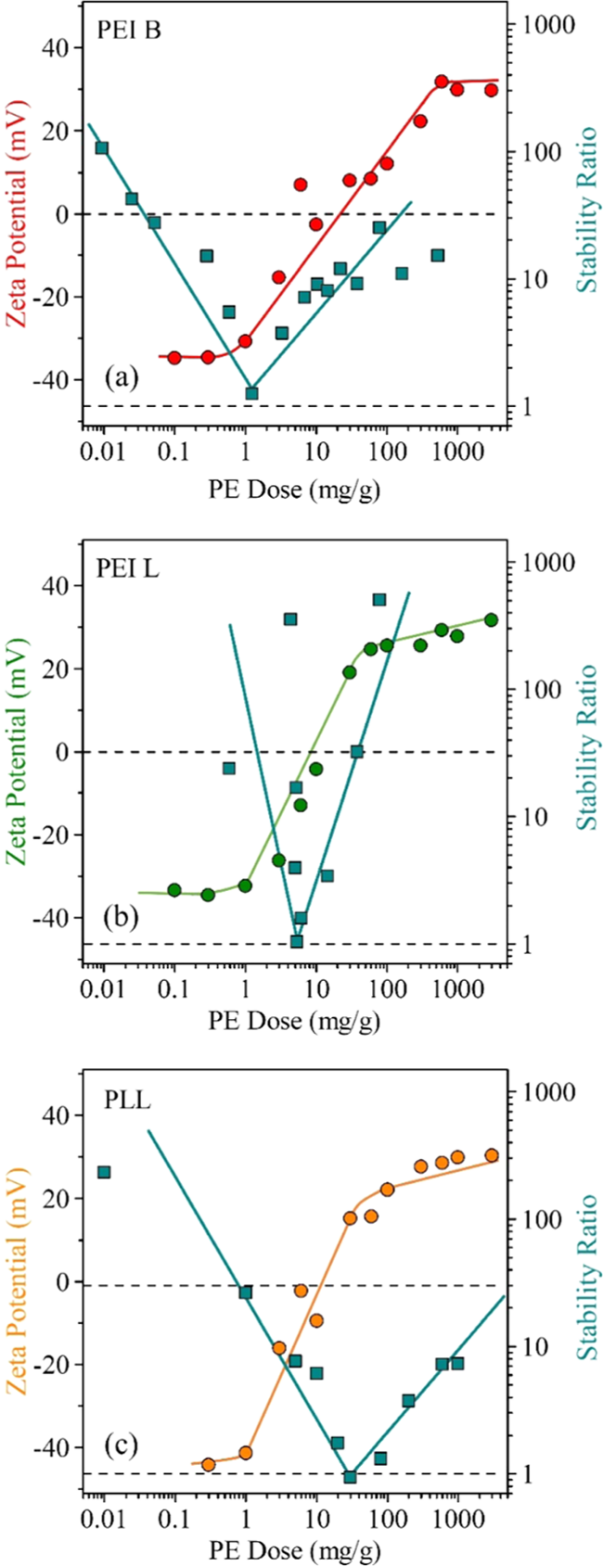
Stability ratios (squares) and ζ-potentials (circles) of BNNS particles in the presence of PEI B (a), PEI L(b), and PLL (c) determined at 0.001 M ionic strength. The solid lines serve to guide the eyes. The mg/g unit refers to mg of polyelectrolyte per one gram of particle.

The ζ-potentials recorded at different PEI B, PEI L, and PLL doses under the same experimental conditions (particle concentration, pH, and ionic strength) are shown in Figure 3a–c, respectively. The same behavior is observed in the case of all three PEs, the points follow similar tendencies. In the beginning, at low PE doses, the values were negative and the ζ-potentials were in the same range as the ones measured in the case of the bare particle at the corresponding ionic strength (Figure 2a). By increasing the PE dose, no significant change can be observed at low concentrations due to the uncompensated charge of the bare particles. However, by further increasing the dose, the ζ-potentials increase, owing to the adsorption of oppositely charged PEs on the BNNS surface. Once a certain PE dose is reached, charge neutralization occurred at the isoelectric point (IEP). This point can be observed at different PE doses, depending on the type of PE. The IEP values were determined as 16.8, 13.0, and 18.7 mg/g in the case of PEI B, PEI L, and PLL, respectively. The reason for the deviations in these values is the difference in line charge density and in the molecular mass of the PEs investigated. However, these differences are not significant.

The adsorption continues beyond the IEP and charge reversal occurred at high PE concentrations. Similar behavior was observed in other particle–polyelectrolyte systems earlier.^28,29,51,52^ The driving forces for such behavior are electrostatic interactions between the oppositely charged moieties and hydrophobic forces between the PE chains and ion correlation forces.^51,53^ Another driving force is the entropy gain due to solvent release upon adsorption.^28,54^ Finally, the values of the ζ-potentials increased until reaching the adsorption saturation plateau (ASP). The onset of the ASP corresponds to the maximum amount of PE that can be adsorbed on the particle surface; it is the dose needed to fully coat the BNNSs.^55^ The excess PEs remain dissolved in the bulk. A slight increase in the mobility values after the onset might be a result of measuring not only the mobility of the coated particles but also the remaining free PEs in the solution.^52^ The ASP values were determined around 300 mg/g in all three cases within the experimental error.

The resulting BNNS–PE complexes are highly enriched in positive charges because of the charge reversal process confirming that the PEs were successfully adsorbed on the surface. It is worth noting that PEI B, in contrast to PEI L, shows slightly different behavior that can be the result of steric hindrance caused by the functional groups. The positive value of the ζ-potential can be used as an indicator of the successful coating of nanoparticles with both PEI^18,23^ and PLL.^56^ When the absolute ζ-potential value is above 30 mV, the dispersions might be considered stable.^57^ PEs may increase hydrophilicity, which is essential to ensure good water dispersibility.^24^ The above-detailed behavior of charge neutralization followed by charge reversal is a common phenomenon and has been reported for different nanoparticles, including BN materials.^28^ The determined ASP values were used to prepare PE-coated BNNS particles to be used in further investigations discussed later.

The aggregation properties and the stability of the particles were also assessed while varying the PE dose in time-resolved DLS measurements under the same experimental conditions as in the ζ-potential study, to directly compare the tendencies in the experimental data obtained. The measured stability ratio values are shown in Figure 3a–3c for PEI B, PEI L, and PLL-coated BNNSs, respectively. Very similar trends were observed with all PEs. Accordingly, the dispersions were stable at low and high PE doses indicated by the high (or not even measurable) values of the stability ratios. However, in the middle region, a minimum in the stability ratios can be observed corresponding to rapid particle aggregation indicated by stability ratios close to unity. Although such an aggregation mechanism was never reported for BNNS–PE systems in the past, it resembles other colloidal or nanoparticle dispersions in the presence of oppositely charged PEs.^28−30,51,52^

One can note that the fast aggregation regime was located around the IEPs. In this region, the dispersions are unstable and the particles rapidly aggregate because the surface charges are neutralized; thus, repulsive electrical double layer interactions are absent and the attractive van der Waals force predominates in line with DLVO theory. Besides, the aggregation regime is notably narrower for the PEI L system compared to the others. This is due to the fact that only half of the amino groups of PEI L are ionized under this experimental condition.^58^ Therefore, PEI L forms a homogeneous layer on the surface, owing to the reduced repulsion between its charged functional groups^59^ leading to the absence of additional (non-DLVO) steric or electrostatic interactions,^60,61^ which may occur for PEI B and PLL.

### Verification of the BNNS Coating

**Figure 4:**
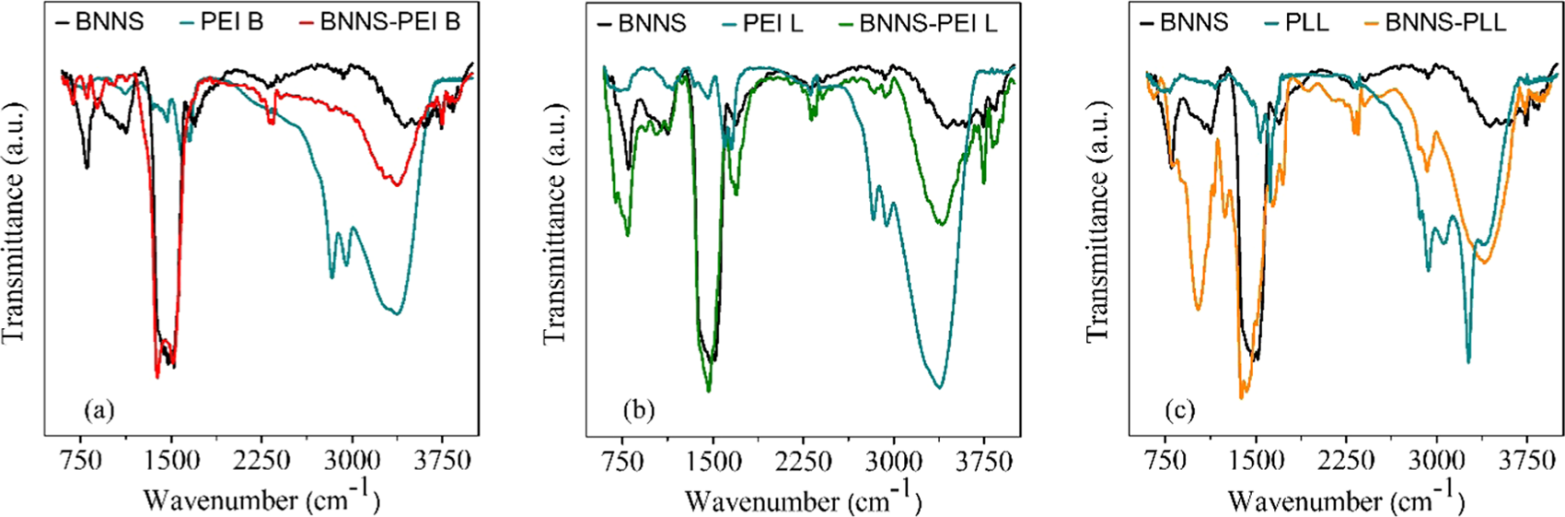
FTIR spectra of bare BNNS particles (black lines), BNNS coated with PEI B (a), PEI L (b), and PLL (c) (red for PEI B, green for PEI L, and yellow for PLL) along with the spectra of the free polyelectrolytes (blue lines).

To further verify the successful functionalization of the particles with PEs, FTIR spectra were recorded (Figure 4). The spectra of noncoated, PE-coated BNNSs, and PEs alone are presented to compare them. In all cases, the spectra of the coated nanoparticles contain the characteristic peaks originating from bare BNNS at 802 and 1481 cm^–1^, which are attributed to the out-of-plane B–N–B bending vibrational mode and the in-plane B–N stretching vibration mode, respectively.^17,37^ The presence of these bands indicates that PEs do not affect the structural characteristics of BNNS.

Besides, the peaks originating from appropriate PEs are also visible on the coated particles’ spectra. PEI B and PEI L both show absorption bands around 1600 cm^–1^ that correspond to the symmetric and asymmetric vibrations of the N–H bond.^37^ In the case of BNNS-PEI B (Figure 4a), these bands overlap with the one of BNNS; however, in the case of PEI L (Figure 4b), they are more separated from the BNNS peak located at 1690 cm^–1^. The bands at 2840 and 2950 cm^–1^ are attributed to the methylene group (C–H) vibrations of PEI;^17^ these are not clearly visible on the BNNS-PEI B composite spectrum but are obvious on the BNNS-PEI L spectrum. A broad peak around 3400 cm^–1^ is also present and is assigned to the characteristic adsorption of amines, N–H asymmetric stretching vibration,^62,63^ for both linear and branched PEI.

In Figure 4c, the FTIR spectrum of the PLL-coated BNNS is shown. Slightly shifted peaks at 1641 and 1720 cm^–1^ can be attributed to peaks at 1543 and 1645 cm^–1^ on the neat PLL spectrum. These bands arise from the amide II vibrational modes of the peptide group and the amide I C=O group stretching mode,^64−66^ respectively. The 2853 and 2923 cm^–1^ adsorption bands originate from the CH_2_ stretching mode of PLL.^65^ A broad peak can be observed in the range from 3050 to 3620 cm^–1^ assigned to the amine (NH_2_ and −NH−) group stretching vibrations.^66−68^ The above results unambiguously confirm the successful functionalization of the BNNSs with oppositely charged PEs.

### Salt-Induced Aggregation

Given the fact that the application of the PE-functionalized BNNS particles is foreseen in liquid media containing electrolytes, the resistance against salt-induced aggregation was tested in a wide range of NaCl concentrations. The nature of the interparticle forces was studied through the effect of ionic strength on the aggregation properties of PE-coated BNNS particles by systematically increasing the concentration of NaCl in the dispersions. The particles were coated with the predetermined quantity of PEs, i.e., a dose corresponding to the onset of ASP was used, which was determined to be 300 mg/g for all systems.

**Figure 5:**
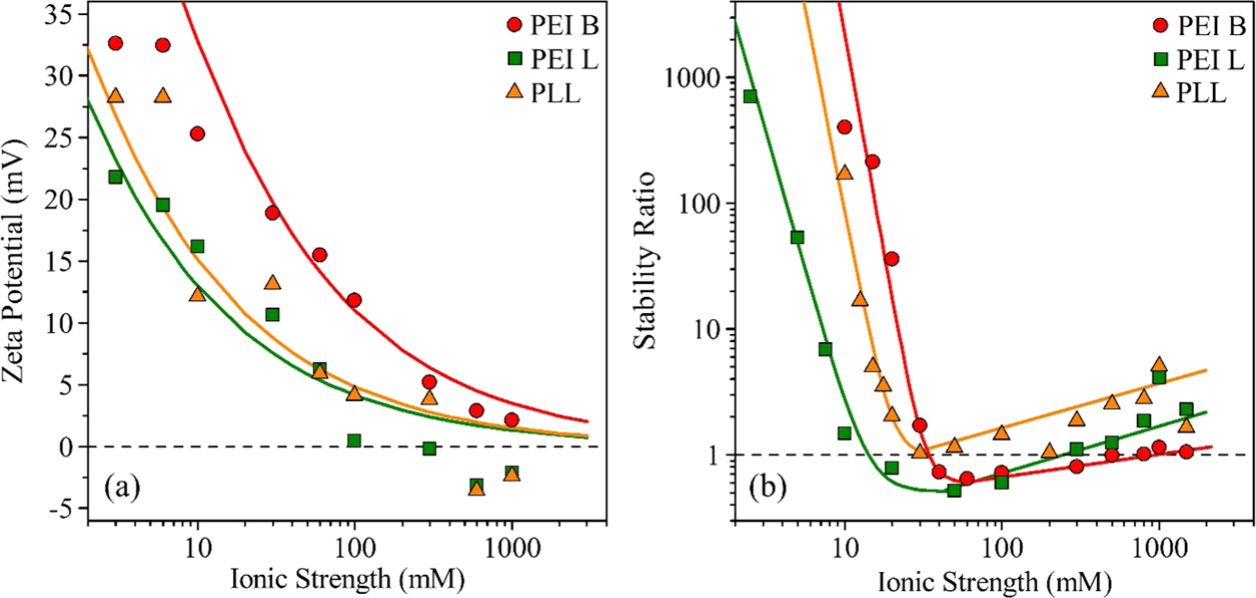
(a) ζ-potentials of BNNS particles coated with PEI B (circles), PEI L (squares), and PLL (triangles). (b) The corresponding stability ratios. The solid lines in (a) were calculated using eq 6, whereas eq 8 was applied in the case of (b).

First, the charging properties were assessed. The ζ-potentials decreased with the increase of the ionic strength in the case of all PEs studied; the results are presented in Figure 5a. This trend is due to charge screening by the dissolved salt constituents.^28^ The ζ-potentials remain negative or close to zero within the experimental error in the entire ionic strength range investigated. A more rapid decrease can be observed in the case of PEI B, while PEI L and PLL show a very similar tendency.

In Figure 5b, the stability ratios calculated according to eq 3 at different ionic strengths are presented. At low ionic strengths, high stability ratios were determined corresponding to the slow aggregation regime. Unstable dispersions can be observed as the ionic strength increases above the CCC values, where the stability ratio is close to unity. The determined CCCs differ for different PEs. The differences originate most likely from the different magnitudes of the surface charge, being the highest in the case of PEI B (8 mC/m^2^), and the lowest for PEI L (1.5 mC/m^2^); for PLL, it was found to be 3 mC/m^2^. Therefore, the calculated CCC values are 30, 20, and 12 mM for PEI B, PLL, and PEI L, respectively. A similar relation between charge densities and CCC values was established with BNNS in the presence of mono- and multivalent salts.^43^

The reason for the above tendency is that the electrical double layer forces are stronger for particles of a higher surface charge; thus, the amount of NaCl necessary to screen such a charge is higher, giving rise to higher CCC. However, at lower surface charge densities, the same effect can be achieved at lower ionic strengths. The shapes of the stability ratio versus ionic strength plots are similar, indicating that the applied PEs induced the same aggregation mechanism, which resembles the one predicted by DLVO theory.

Nevertheless, the slight increase in the stability ratios at high salt levels is not foreseen by the DLVO model, indicating the presence of additional stabilizing forces. One possibility is the increase of the steric stabilization effect,^17,60,61^ which originates from the overlap of the adsorbed PE chains upon the approach of another particle; hence it can further stabilize the particles leading to higher stability ratio values. This scenario can be further confirmed by the fact that PE chains adsorbed on surfaces usually swell at high ionic strengths,^55,69^ and such an extended interfacial structure enhances steric interactions. However, this assumption was not supported by direct experimental evidence in the present study.

## Conclusions

The colloidal behavior of the synthesized BNNS particles was investigated in the presence of three types of polyelectrolytes, namely, PEI B, PEI L, and PLL. PEs adsorbed on the oppositely charged particle surface due to various interactions, but the major forces were of electrostatic origin. The adsorption of PEs resulted in charge neutralization followed by charge reversal. At a certain PE dose, an adsorption saturation plateau was observed; the PE dose needed to fully coat the particle surface was determined by its onset. This dose was 300 mg/g for all three compounds studied. The functionalization was further confirmed by FTIR spectroscopy.

The salt-induced charging and aggregation properties of the coated nanoparticles were studied to get an insight into their behavior in dispersions with elevated ionic strengths. Very similar features were observed in the case of all three PEs; charge screening led to the compression of the electrical double layer, thus weakening the repulsive forces between the particles. The CCCs were determined and they decreased by decreasing the charge density of the PE-coated particles. The PEI B-coated BNNS was the most stable, i.e., showed the highest CCC, due to additional steric stabilization effects. The aggregation mechanism in general could be described in terms of DLVO-type and steric interparticle forces.

The knowledge gained in this study allows the prediction of the aggregation behavior of BNNS particles in the presence of PEs and simple salts. As the application of BNNS usually takes place in liquid media, these findings will attract considerable attention in the scientific and technological communities, wherever stable or unstable BNNS dispersions are desired for certain applications. Accurate knowledge of the stability of such dispersions is highly needed; therefore, the results on the present BNNS–PE systems may contribute to develop new applications.
